# Sleep Alterations in the Population of the Metropolitan Area of Mexico and Their Association with Lifestyle Changes During COVID-19 Confinement

**DOI:** 10.3390/clockssleep7010006

**Published:** 2025-02-17

**Authors:** María del Rosario Ayala-Moreno, Paola Andrea Martínez-Serrano, Montserrat Alheli Melgarejo-Gutiérrez, Alma Rosa Hernández-Mondragón, Azucena Martínez-Basila, Araceli Martínez-Coronado, María José Losana-Valencia, Esther Vargas-Medina, Eloisa Colín-Ramírez, Adriana Benítez-Rico

**Affiliations:** 1 Research Group Study of Non-Communicable Diseases and Aging, La Salle University, Mexico City 06140, Mexico; paola.m.serrano@gmail.com (P.A.M.-S.); maria.losana@lasallistas.org.mx (M.J.L.-V.); esther.vargas@lasalle.mx (E.V.-M.); adriana.benitez@lasalle.mx (A.B.-R.); 2 Laboratorio de Neurobiología del Sueño y Metabolismo, Facultad de Medicina, Universidad Veracruzana, Veracruz 91010, Mexico; momelgarejo@uv.mx; 3 Research Group in Management and Leadership for Innovation and Quality of Education, La Salle University, Mexico City 06140, Mexico; aracne6060@gmail.com; 4 Research and Metabolism Center, Mexico City 03840, Mexico; a.martinezb@lasallistas.org.mx; 5 Department of Biological and Health Sciences, Metropolitan Autonomous University, Mexico City 04960, Mexico; nutcheli@hotmail.com; 6 Dirección de Nutrición, Instituto Nacional de Ciencias Médicas y Nutrición Salvador Zubirán, Mexico City 14080, Mexico; eloisa.colinr@incmnsz.mx

**Keywords:** sleep alterations, sleep quantity, sleep quality, sleep hygiene, COVID-19 confinement, lifestyles

## Abstract

Home confinement due to Coronavirus Disease 2019 (COVID-19) led to lifestyle changes that increased sleep disturbances, particularly in areas with higher infection and mortality rates. This study is a retrospective study based on data collected through an online survey conducted during the COVID-19 confinement. It aims to analyze changes in sleep quantity and quality and their association with lifestyle changes in the metropolitan area of Mexico City. A total of 899 adults from this area completed an online questionnaire between June 2020 and February 2021. This study assessed sleep quantity, sleep quality, insomnia symptoms, and lifestyle changes during the confinement period. Results showed that sleep quantity increased (7.10 ± 1.37 vs. 7.43 ± 1.42 h, *p* < 0.0001), with more participants, especially young adults and women, reporting later bed and wake-up times. The Pittsburgh Sleep Quality Index increased by 1.4 units, with poor sleep quality associated with lifestyle during confinement. Insomnia symptoms, sleep latency, and poor sleep quality also increased, particularly in women. Males and those without chronic comorbidities were less likely to experience poor sleep quality, while tobacco use and later bedtimes increased this risk. This study concludes that, while sleep quantity increased, sleep quality declined, particularly among young adults, women, and those with unhealthy lifestyles. These findings could guide sleep health initiatives tailored to specific lifestyle changes in different population groups.

## 1. Introduction

Mexico was considered one of the countries with the highest accumulated cases of Coronavirus Disease 2019 (COVID-19) in Latin America [1]. The partial lockdown strategy aimed at reducing virus transmission prolonged the epidemiological alert and home confinement period, especially in areas with high population density and elevated infection and mortality rates due to COVID-19, such as the metropolitan area of Mexico. Social isolation and reduced mobility changed lifestyles and thus affected the chronicity of physiological processes including sleep [2,3,4], which is vital for a person’s overall health and well-being. The lack of sleep or poor sleep quality can negatively affect immune responses [5], mental health [6], and cardiometabolic health [7]. The social, economic, and academic challenges arising from the pandemic, as well as the fear of catching the disease and the lack of treatment, heightened psychological stress with symptoms that are associated with sleep disturbances [3,8,9,10,11]. Stress associated with confinement was also linked to sleep disturbances, such as bruxism [12], which was also associated with poor sleep quality [13]. The first studies on pandemic-related sleep disturbances were published between February and March 2020 by countries that were implementing maximum mobility restriction at the time. These studies reported a 12% to 70% increase in insomnia, especially in front-line healthcare workers (doctors, nurses, and caregivers) [14,15,16,17], as well as increased nocturnal awakenings and prolonged sleep latency in COVID-19 patients or their direct caregivers [18]. Other studies have described sleep disturbances in non-infected populations that are still confined to their homes and experience the stress of isolation, changes in their daily routine and dietary habits, decreased physical activity, and increased screen time for work. These characteristics, together with reduced exposure to natural light, appear to desynchronize the central circadian clock, affecting sleep duration and quality [4,18,19].

Despite the differences in pandemic management strategies, in the degree of restrictions during lockdown, in different behaviors according to sociocultural environments, and in research time frames, meta-analysis studies report a global increase of 35.7% (95% CI, 29.4–42.4%) in sleep impairment [9], emphasizing a decrease in sleep quality and an increase in insomnia [8]. Despite this global context, in Mexico, a nationally representative analysis did not report significant changes in the sleep duration of the adult population, and other sleep indicators such as quality or the factors determining the quantity and quality of sleep were not explored [20]. In addition, it is relevant to mention that these results did not derive from a validated survey for analyzing sleep variables. Therefore, a specific analysis of sleep disruptions during the pandemic is necessary, especially in population sectors of cities with high infection and mortality rates, as they have implemented extended confinement periods, and their populations have been exposed to greater stress levels, social isolation, and lifestyle changes, which are determinants of sleep quantity and quality. Despite reports of sleep disturbances in various populations during the pandemic, it is crucial to investigate these alterations within Mexican populations and share these findings. This will help in developing sleep hygiene programs tailored to future health emergencies, ensuring that they are more consistent with the specific needs and environment of these regions. It is important to personalize such programs because the specific environmental factors of a population may condition the circadian cycles in different ways, resulting in singular adaptive response patterns related to the environment [21,22]. This work aims to analyze the alterations in sleep quantity and quality and their association with lifestyle changes in a population from the metropolitan area of Mexico City. This retrospective study is based on data collected through an online survey conducted during the COVID-19 confinement.

## 2. Results

### 2.1. Characteristics of Participants and the Result of the Questionnaire Survey

A total of 1475 surveys were received, of which *n* = 329 were removed because respondents were not from the metropolitan area of Mexico City and *n* = 247 because they were not completely answered. Finally, we included a total of 899 surveys in this analysis, 674 of which were female (74.97%) and 225 (25.03%) males. All respondents had access to a computer, tablet or smartphone, and connectivity to use social networks and WhatsApp to answer the survey. The mean age was 39.92 ± 12.40 years old, 12.01% of respondents ranged from 18 to 25 years old, and 87.98% were adults (26–64 years old) and older adults (≥65 years old). The respondents were residents of the different regions of the metropolitan area of Mexico City (25.76% from the northern zone, 29.26% the western zone, 4.72% the eastern zone, and 20.52% from the southern zone), and 19.74% were from Mexico City’s surroundings. Regarding educational level, 1.63% reported having elementary school and/or middle school, 7.41% high school, 50% university level, and 40.96% graduate level. Finally, the presence of comorbidities with a known diagnosis (obesity, arterial hypertension, diabetes, dyslipidemias, and cancer) was reported by 40.60% of the respondents.

Table 1 shows the general sleep variables before and during home confinement, stratifying respondents by sex. Respondents experienced an increase in sleep quantity (0.33 h, *p* < 0.0001) and sleep latency (13.46 min, *p* = 0.0001) during the confinement period. One highlight is that sleep latency showed a large effect size (*d* = 0.91) on the overall population, and especially on females. More respondents reported a later bedtime and waking up later (*p* = 0.0001) between 3 and 7 days/week, especially females (*p* < 0.017). Additionally, more young people (18–25 years old) reported having a later bedtime than usual during confinement (37.95% vs. 70.37%), followed by adults 26–64 years old (31.11% vs. 58.18%) and older adults ≥ 65 years old (19.0% vs. 61.9%), with no differences between these age groups.

**Figure 1:**
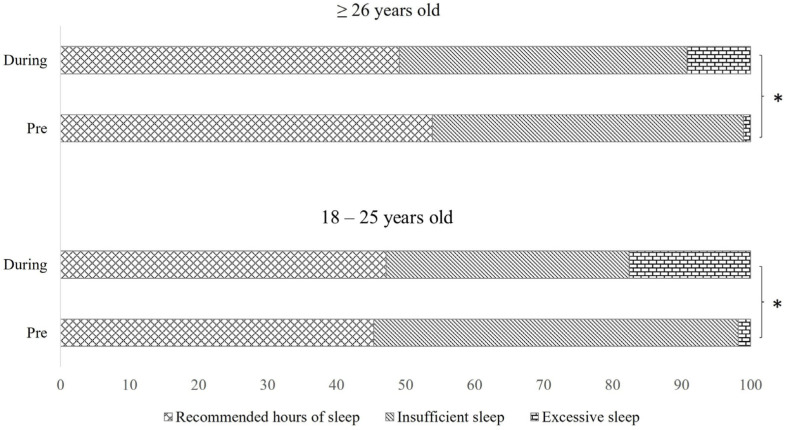
Comparison of adherence to sleep quantity recommendations by age group between pre-COVID-19 and during-COVID-19 periods. Data are expressed as the percentage of participants. Recommended sleep hours refer to participants who met the recommended hours of sleep for their age group. Insufficient sleep refers to sleeping fewer hours than recommended, while excessive sleep refers to sleeping more hours than recommended. Recommendations are based on the National Sleep Foundation’s (NSF) guidelines for each age group (18–25 years, 26–64 years, and ≥65 years). * Indicates a statistically significant difference in the percentage of participants with excessive sleep between the pre-COVID-19 and during COVID-19 periods (*p* < 0.035, McNemar test).

The analysis of adherence to sleep quantity recommendation by age group showed that nearly half of the respondents reported having insufficient sleep (fewer hours than recommended for their age) pre-COVID-19 confinement regardless of age. Additionally, before the pandemic, an exceptionally low percentage of participants slept excessively, while, during confinement, more respondents reported having excessive sleep (18–25 years old, 1.9% vs. 17.6% and ≥26 years old, 0.4% vs. 9.2% (Figure 1).

### 2.2. Pittsburgh Sleep Quality Index (PSQI) Survey Score Pre-COVID-19 and During COVID-19

Regarding sleep quality, the PSQI increased 1.4 units (*p* = 0.0001). These changes are accompanied by a medium effect size in the total population (*d* = 0.47), which is maintained in the female population (*d* = 0.56). Additionally, all the components of the PSQI showed higher and significant values during the contingency period, with sleep latency and habitual sleep efficiency highlighted as variables that exhibited a medium effect size. Overall, these results indicate an increase in acute sleep disturbances. Note that sleep duration, as a component of the PSQI, decreased during confinement (1.47 vs. 1.32, *p* < 0.0001); however, because of PSQI’s scoring guidelines, this means that sleep duration increased. In addition, alterations in sleep latency, subjective sleep quality, habitual sleep efficiency, as well as sleep disturbances, daytime impairment, and insomnia symptoms, were greater in women than in men (*p* ≤ 0.006). Additionally, a medium effect size was observed for the prevalence of insomnia and a large effect size for excessive sleepiness (Table 2).

**Figure 2:**
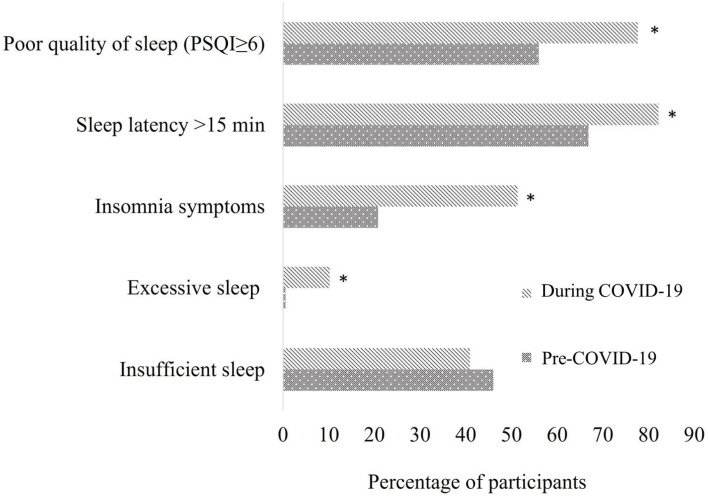
General effect of confinement on sleep disturbances. A Pittsburgh Sleep Quality Index (PSQI) score ≥6 indicates poor sleep quality [25]. Insufficient sleep and excessive sleep are classified based on the National Sleep Foundation’s (NSF) sleep recommendations for each age group. * Indicates a significant difference in the overall study population between pre-COVID-19 and during COVID-19 periods (*p* < 0.01, McNemar test).

To sum up, the effect of home confinement on sleep disturbances highlights that the percentage of respondents with excessive sleep, prolonged sleep latency (>15 min), poor sleep quality, and insomnia symptoms increased significantly (Figure 2).

### 2.3. Lifestyle During Confinement

The prevalence of poor sleep quality in respondents was dependent on lifestyle during confinement. Respondents reported worse sleep quality on days with minor sunlight exposure, with minor physical exercise, and with more time spent using electronic devices. Additionally, respondents who did activities, which included physical work during the day, such as exercising, house cleaning, playing with children, dancing, gardening, and others like breathing exercises or meditation for emotional care reported a lower frequency of poor sleep quality. In contrast, the respondents who showed a higher prevalence of poor sleep quality did activities without body mobility including playing board games, drawing, painting, reading, making crafts, using social networks, completing online workshops or courses, and watching movies (Table 3). Complementary to these results, logistic regression analysis for the association between environmental factors and poor sleep quality showed that being male and not presenting chronic comorbidities decreased the risk of having poor sleep quality (*p* = 0.045), while tobacco use (OR 1.68, 95% CI 1.050–2.688) and having a later bedtime significantly increases this risk (OR 2.98, 95% CI 1.485–5.996). It is especially notable that these last two environmental factors present a small and medium effect size, respectively (Table 4). Other variables, such as general aspects of life dynamics during confinement (e.g., living with someone diagnosed with COVID-19 or being diagnosed with COVID-19), did not significantly explain the increased poor sleep quality, although these variables showed a small effect size.

## 3. Discussion

Few studies have analyzed the relationship between sleep disturbances and lifestyle changes derived from Mexico’s quarantine period, specifically in areas with high infection and mortality rates due to COVID-19. Our results show that sleep disturbances were not a confinement-specific problem, as almost half of the respondents experienced insufficient sleep, prolonged sleep latency, and poor sleep quality before lockdown. Moreover, 20.80% of respondents stated having insomnia symptoms before quarantine, which is consistent with the influence of environmental and lifestyle factors on sleep, and similar results have been found in studies performed in other cities [26]. It is worth mentioning that comparing our findings with those of previous studies in Mexican populations is complicated due to the use of different instruments to assess sleep variables. However, the results of this study are consistent with the national prevalence of insomnia (20.80% vs. 18.80%) and sleep duration (7.10 ± 1.37 h vs. 7.6 ± 3.0 h), but they differ from results on sleep insufficiency in Mexico City (38.1%, 95% CI, 32.2–44.3), perhaps because the cut-off point was different (<7 h/day) [27]. In this regard, our results were based on the NSF’s sleep insufficiency criteria (<6 h/day). Despite the stricter cut-off point, we found that 52.77% of respondents in the 18–25 age group (95% CI, 49.44–55.96%) and 46.36% of those in the 25–64 age group (95% CI, 43.01–49.70%) experienced sleep insufficiency before the quarantine period. Furthermore, sleep deficiency has been considered a public health epidemic since before the COVID-19 outbreak, and studies had already detected an increased prevalence of this sleep disturbance [28,29,30]. This global trend of sleep insufficiency changed during quarantine [31], as a significant increase in sleep duration was reported. Although a previous study in Mexico found only a minor change in sleep duration [20], our results are like those of other studies in cities with large populations like in the US [31,32], Europe [32,33], and Singapore [34]. The increase in sleep duration has been explained by the decrease in mobility due to remote working and the closure of leisure and entertainment venues [31], which may reduce social jet lag and increase the amount of time available for rest [33].

According to other studies, aspects of sleep that were affected during the pandemic include later bed and wake-up times, prolonged sleep latency, and poor sleep quality [8,31,34,35]. These findings were also observed in our study and are particularly notable in the case of women (with significant results and a small effect size). These shifts in sleep patterns may be explained by the fact that screen time increased for work, school, and social purposes [36,37,38]. In this sense, our study found that 45.5% of the participants used electronic devices 4 to 8 h/day (data not presented). Consistent with the analysis of the prevalence of poor sleep quality, it was higher in individuals who used electronic devices more frequently throughout the week (*p* = 0.018). Moreover, Priego-Parra et al. (2020) reported that 62.7% of their population presented internet addiction during lockdown [35]. This alteration was more evident in young adults, who have also experienced a significant emotional impact from social isolation [35,39,40,41], making them a particularly vulnerable sector of the population. These results support the importance of maintaining consistent sleep schedules during public health emergencies, as disrupted sleep is considered a risk factor for poor sleep quality, especially when these disruptions occur more than three days a week. This result is notable due to the medium effect size. The behavior associated with the desire to sleep is determined by melatonin [42,43]. The mainly nocturnal and regular secretion of this hormone stabilizes the sleep–wake cycle [42]. Therefore, lifestyle changes, such as decreased exposure to sunlight and overexposure to artificial light may alter melatonin pulses, generating a chrono-disruption that would explain the prolonged sleep latency and irregular sleep schedules reported in the survey. Chrono-disruption may also exacerbate metabolic disturbances [21,42,43,44,45], especially during extended periods of home confinement, as melatonin is involved in the regulation of food intake and energy metabolism [45].

The increase in insomnia symptoms and sleep quality disruptions may be explained not only by chrono-disruption but also by the stress generated by the COVID-19 pandemic, as suggested in other studies [8,46,47,48]. Although our study did not analyze the psychological effects of the pandemic, Priego-Parra (2020) found a 51% increase in anxiety and up to 86% increase in depression during the initial weeks of lockdown in Mexico [35]. These effects derived from social isolation, employment and academic uncertainty, job loss, and fear of contagion [8,17,46,47,48,49]. According to our findings, insomnia symptoms showed a considerable increase during the confinement. However, women were particularly affected by insomnia during the quarantine. This is in line with other studies that have reported that sleep duration, poor sleep quality, and sleep latency were greater, and insomnia symptoms more frequent, in women than in men during this period [8,33,35,41]. These disruptions are likely associated with findings that women have experienced greater psychological distress during quarantine because domestic work, childcare, and family caregiving responsibilities fall on their shoulders. In addition to these responsibilities, women have also had to maintain their roles as either partial or sole providers for their families [47,48,49,50]. Our results reinforce the need to consider women among the groups most vulnerable to sleep disturbances during health emergencies.

For their part, the small group of adults over age 65 slept well before the pandemic but showed deterioration in sleep duration (more or less sleep than recommended for their age), increased symptoms of insomnia, and poor sleep quality during the confinement period. These disruptions could have derived from the emotional decline linked to socially isolated, vulnerable populations [51,52]. Our group of older adults was too small to adequately analyze the results, so these were only included as part of the overall description of the data. Further studies in this sector of the population are needed to provide them with adequate support in future health emergencies.

Our results suggest that poor sleep quality is higher among people that tend to engage in sedentary activities consistent with previous reports [53,54]. Therefore, spending time on daily activities with greater and continuous muscle work may be important during confinement. At home, it is possible to conduct low-energy demand routine activities, such as playing with children, cleaning, gardening, and others that may have a significant impact on sleep quality.

It is evident that our study had low male participation in the survey responses (*n* = 225 males vs. *n* = 674 females), which could limit the ability to establish significant sex differences. However, our analysis shows that some individual attributes, such as sex, may influence the risk of poor sleep quality, while other individual and health social factors—such as age or being diagnosed or living with someone who has been diagnosed with COVID-19—did not show a significant risk for poor sleep quality, even though other studies have described this risk [18,41,55]. This is probably because the number of participants reporting having or living with someone with COVID-19 was small in the period when the survey was conducted (*n* = 96, 10.67%). Similarly, reduced physical activity and the consumption of stimulants such as caffeine were not identified as risk factors for poor sleep quality, despite other studies reporting an association with sleep quality, duration, and latency [56,57,58,59]. This discrepancy may be explained by the fact that our study did not thoroughly investigate the amounts of stimulating drinks consumed. Additionally, the survey did not assess the time spent exercising or the type of physical activity performed. In contrast to the above, having healthier habits, such as sleeping the recommended hours per night, not consuming tobacco, and not presenting comorbidities decreased the risk of having poor sleep quality, reinforcing the importance of adopting a healthier lifestyle to decrease the negative impact of confinement.

Analyzing sleep variables may be complex because the instruments are based on subjective reports of well-being and daytime functioning [60]. In addition, other aspects, such as the different seasonal periods during which the surveys were completed and that could influence aspects of sleep quantity and quality, were not considered in this study. However, it is relevant to include the population’s perception of sleep quality deterioration, an aspect that may impact immune function [36,37,38]. Proper sleep quantity and quality support immune and inflammatory homeostasis, improve the immune response to vaccination, and favor recovery from an infectious event [5,61,62], so good sleep may contribute to the prevention of COVID-19 infections [63]. It is essential to promote sleep hygiene as part of a healthy lifestyle, especially during health emergencies. The results highlight the importance of providing strategies to help maintain a better lifestyle and help the general population to take care of themselves and their families during health emergencies, including those populations of metropolitan areas with a substantial risk of being infected and those with reduced mobility, in which sleep problems may be exacerbated. The COVID-19 pandemic has had a major impact on global sleep health.

Finally, based on the findings of the present study, we recommend addressing specific aspects of sleep hygiene that could help support future public health emergencies involving home confinement:

(1) Maintain a consistent sleep routine by adhering to regular sleep schedules, particularly at bedtime.
(2) Keep windows open to allow natural daylight into your home, and ensure it is visible.
(3) Aim for moderate sunlight exposure (1–1.5 h in the morning or afternoon).
(4) It is recommended to balance physical activity and sleep. Engaging in low to moderate physical exercise, reducing screen time, and dedicating time to personal interests or family activities promote a mild but consistent increase in physical activity.
(5) Implement stress management techniques, which can be integrated into healthcare programs aimed at promoting better sleep hygiene as a strategy to reduce negative health impacts.
(6) Within the family, agree on an equitable distribution of responsibilities to reduce the workload on women or caregivers.
(7) For young people, it is particularly important to involve them in family interaction activities or physical exercise to reduce time spent on cell phones, tablets, or computers.

## 4. Materials and Methods

### 4.1. Participant Recruitment

Sample size was estimated based on the overall population living in Mexico City and the State of Mexico, where participants were recruited. According to the National Institute of Statistics and Geography (INEGI), in 2020, there were 18,299,362 adult (20 years and older) residents in these two entities. Using the formula for finite populations, with a 95% confidence level, and a 5% margin of error, a minimum of 385 participants was estimated. Assuming a 20% non-response rate, a total sample of 462 participants to be surveyed was rendered.

Participants (male and females, >18 years old) were recruited using a convenience snowball-sampling method. Individuals were invited through social media such as Facebook, Instagram, e-mail, and WhatsApp.

As part of a diagnosis phase of the study “Analysis and Attention to Health Risk Factors in the COVID-19 Contingency” (ANFARIS study), participants were asked to fill out an electronic self-administered questionnaire. The La Salle University Research Committee approved this protocol (emergent projects COVID-19, with approval number ID34-2020), and it was developed in accordance with the principles of the Helsinki Declaration (1964) and its later amendments. Participation was anonymous, and respondents only accessed the items if they previously gave an informed consent.

### 4.2. Survey Characteristics

The survey was developed with Google Forms and was available from 3 June 2020 until 18 February 2021, which were the months with the highest infection rates due to the pandemic in Mexico, with varying mortality records between 5000 and 7000 deaths per epidemiological week [64]. The survey contained 186 items concerning sociodemographic information (sex, age, metropolitan area of residence, education level, house type) and the presence of comorbidities with known diagnosis (obesity, arterial hypertension, diabetes, dyslipidemias, and cancer), lifestyle changes, and 44 items related to sleep quantity, sleep quality, and insomnia symptoms. Respondents were instructed to self-report sleep variables prior to confinement by recording their perception of each variable, referencing the month preceding the start of the confinement period (i.e., the period before the official stay-at-home announcement issued by the federal government on 23 March 2020). Additionally, for responses given during the confinement period, all participants referred to the last month of confinement, considering that, by the time the survey was conducted, everyone had been in confinement for at least three months.

Before sharing on social networks, the reliability and consistency of the items of the instrument were insured by determining the Cronbach’s alpha values for each item in a sample of 100 participants [65,66]. For each item, Cronbach’s alpha values in the range of 0.6 to 0.8 were considered acceptable, and the reliability level obtained of 0.81 allowed us to conclude that the study sample presented a high degree of heterogeneity.

### 4.3. Sleep Quantity and Sleep Quality

The Pittsburgh Sleep Quality Index (PSQI), translated into Spanish [67] and validated for the population of Mexico [60], was used to determine the quantity and quality of sleep. Subjects with a PSQI global score ≥6 were classified as having poor sleep quality according to Buysee et al. (1989) [25].

Sleep quantity was calculated as the difference between bedtime and wake-up time minus the time taken to fall asleep (sleep latency in minutes). Sleep was insufficient (sleep duration less than recommendation) or excessive (sleep duration higher than recommendation) according to the National Sleep Foundation’s (NSF) recommended sleep times for each age group (18–25 years, 26–64 years, and ≥65 years) [68], as specified in results.

### 4.4. Insomnia

The following question was used to identify the presence of insomnia symptoms: “In the last three weeks, have you had trouble falling asleep and/or staying asleep, or woken up earlier than you desired?” If these symptoms were present for ≥3 days/week, subjects were considered as having insomnia [27].

### 4.5. Lifestyle Changes Related to Sleep Disorders

Changes in eating habits, physical activity, and lifestyle were explored for their association with sleep. Specific changes included the frequency of exposure to sunlight, physical exercise, time spent using electronic devices, the consumption of stimulant drinks such as alcohol, caffeine, or black tea, tobacco use, and kind of hobby during confinement.

### 4.6. Statistical Analysis

Normally distributed variables are presented as means and standard deviations. Student’s *t*-test or ANOVA was used for analysis, and the effect size was calculated using Cohen’s *d*, classified as small (*d* = 0.2), medium (*d* = 0.5), large (*d* = 0.8), and very large (*d* = 1.3) [23,24]. Categorical variables are presented as absolute and relative frequencies and were compared by the χ2 or McNemar test. Univariate and multivariate binary logistic regression analyses were performed to study the association between lifestyle variables and poor sleep quality. Odds ratio (OR) and 95% confidence intervals (CIs) were estimated. For the OR, the effect size was considered small (OR = 1.5), medium (OR = 2), or large (OR = 3) [23].

Data were analyzed with SPSS statistics software 27.0. In all cases, *p* < 0.05 was considered statistically significant.

## 5. Conclusions

These findings highlight the increase in sleep duration and sleep disturbances including sleep latency, insomnia symptoms, and the deterioration of sleep quality among the metropolitan area of Mexico, a geographical area with high population density and higher infection and mortality rates during the confinement period. Chrono-disruption is a factor that increased the risk of poor sleep quality and was evident mainly in women and young adults. Aspects related to a healthy lifestyle, such as sleeping the recommended hours per age, avoiding delayed sleep timing, not consuming tobacco, and integral healthcare to reduce comorbidities, may decrease the risk of poor sleep quality. Poor sleep quality, sleep delay reflected in bedtime, and the higher prevalence of insomnia symptoms stand out particularly for their medium and high impact on female, making it a highly vulnerable group to be addressed during future health emergencies.

## Figures and Tables

**Table 1:** Description of general sleep variables pre and during COVID-19 period stratified by sex.

	Pre Mean ± SD	During Mean ± SD	*p* ^1^	Effect Size ^2^ (Cohens´s *d*)
Sleep quantity (h)	6.85 ± 1.43 ^a^	7.40 ± 1.30	0.0001	0.38 *
Male				
Female	7.18 ± 1.34 ^b^	7.43 ± 1.46	0.0001	0.19
Total	7.10 ± 1.37	7.42 ± 1.42	0.0001	0.23*
Sleep latency (min)	25.20 ± 15.16	35.40 ± 20.34 ^a^	0.0001	0.67 **
Male				
Female	24.48 ± 14.72	39.03 ± 21.87 ^b^	0.0001	0.99 ***
Total	24.66 ± 14.83	38.12 ± 21.54	0.0001	0.91 ***
Bedtime later than usual (3–7 day/week)	51 (5.70%)	113 (12.56%) ^a^	0.0001	
Male				
Female	116 (13.00%)	424 (47.16%) ^b^	0.0001	
Total	300 (33.37%)	537 (59.73%)	0.0001	
Waking up later than usual (3–7 day/week)	13 (0.01%)	86 (0.095%)	0.0001	
Male				
Female	39 (0.04%)	315 (35.04%)	0.0001	
Total	52 (5.78%)	401 (44.60%)	0.0001	

Data are presented as the mean ± SD or the number of participants and percentage. ^1^ Indicates the statistical significance between pre-COVID-19 and during COVID-19 periods (Paired *t*-test or McNemar test, as appropriate according to the type of data). Comparisons between sexes are determined with Student’s *t*-test or χ2. Different letters indicate a significant difference when comparing men and women (*p* < 0.05). ^2^ Effect size is expressed as Cohen’s index (*d*) for data presented as mean and SD, with relative effect stratified as * small (*d* = 0.2), ** medium (*d* = 0.5), *** large (*d* = 0.8), and very large (*d* = 1.3) [23,24].

**Table 2:** Description of Pittsburgh Sleep Quality Index (PSQI) components and symptoms of insomnia pre- and during COVID-19 period stratified by sex.

	Pre Mean ± SD	During Mean ± SD	*p* ^1^	Effect Size ^2^ (Cohen’s *d*)
PSQI	6.19 ± 2.93	6.81 ± 3.32 ^a^	0.0001	0.21 *
Male				
Female	6.36 ± 2.96	8.01 ± 3.54 ^b^	0.0001	0.56 **
Total	6.32 ± 2.95	7.71 ± 3.52	0.0001	0.47 *
Subjective sleep quality	1.27 ± 0.74	1.36 ± 0.75 ^a^	0.0001	0.12
Male				
Female	1.25 ± 0.74	1.58 ± 0.81 ^b^	0.0001	0.45 *
Total	1.26 ± 0.74	1.53 ± 0.80	0.0001	0.36 *
Sleep latency	0.97 ± 0.88	1.45 ± 1.01 ^a^	0.0001	0.55 **
Male				
Female	0.99 ± 0.86	1.66 ± 1.01 ^b^	0.0001	0.78 **
Total	0.98 ± 0.86	1.61 ± 1.01	0.0001	0.73 **
Sleep duration	1.61 ± 0.84	1.36 ± 0.91	0.0001	0.3 *
Male				
Female	1.43 ± 0.85	1.31 ± 0.99	0.0001	0.18
Total	1.47 ± 0.85	1.32 ± 0.97	0.0001	0.07
Habitual sleep efficiency	0.03 ± 1.16	0.06 ± 0.023 ^a^	0.001	0.03
Male				
Female	0.03 ± 0.19	0.14 ± 0.35 ^b^	0.003	0.58 **
Total	0.03 ± 0.18	0.12 ± 0.33	0.0001	0.50 **
Sleep disturbance	0.98 ± 0.60	1.12 ± 0.67 ^a^	0.001	0.23 *
Male				
Female	1.11 ± 0.52	1.31 ± 0.59 ^b^	0.0001	0.38 *
Total	1.08 ± 0.55	1.26 ± 0.62	0.0001	0.33 *
Use of sleep medication	0.32 ± 0.72	0.39 ± 0.85	0.0001	0.10
Male				
Female	0.36 ± 0.80	0.49 ± 0.99	0.0001	0.16
Total	0.35 ± 0.78	0.46 ± 0.96	0.0001	0.14
Daytime dysfunction	1.01 ± 0.89	1.07 ± 0.96 ^a^	0.0001	0.07
Male				
Female	1.18 ± 0.91	1.51 ± 0.96 ^b^	0.0001	0.36 *
Total	1.14 ± 0.91	1.40 ± 0.98	0.0001	0.29 *
Frequency of symptoms of insomnia (%)	42 (18.66%)	98 (43.55%) ^a^	0.0001	
Male				
Female	145 (21.51%)	365 (54.15%) ^b^	0.0001	
Total	187 (20.80%)	463 (51.50%)	0.0001	

Data are presented as media ± SD or the number of participants and percentage. ^1^ Indicates the statistical significance between the pre-COVID and during COVID-19 period (t-Student for dependent samples or McNemar test, as appropriate according to the type of data). Different letters indicate a significant difference when comparing men and women (*p* < 0.05). ^2^ Effect size is expressed as Cohen’s index (*d*) for data presented as the mean and SD, with relative effect stratified as * small (*d* = 0.2), ** medium (*d* = 0.5) [23,24].

**Table 3:** Prevalence of poor quality of sleep according to lifestyle during confinement.

Activity or Lifestyle Habit			*p*
Frequency of exposure to sunlight	3–7 day/week 28.7%	0–2 day/week 71.3%	0.056
Frequency of physical exercise	0–2 day/week 71.77%	3–7 day/week 28.22%	0.004
Time spent using electronic devices ^1^	0–3 h/day 34.38%	4–8 h/day 65.61%	0.018
Consumption of stimulant drinks ^2^	Decreased 32.9%	Increased 67.1%	0.43
Tobacco use	Non-smoker 20.34%	Smoker 79.65%	0.016
Kind of hobby during confinement ^3^	With body mobility/meditation 8.08%	Without body mobility 91.92%	0.025

^1^ Including use of TV, computer, tablet, cell phone, and video games. ^2^ Including consumption of coffee, cola drinks, black tea, and cocoa drinks. ^3^ Activities with body mobility included physical exercise, house cleaning, playing with children, dancing, and gardening. Activities without body mobility included board games, drawing, painting, reading, crafts, use of social networks, online workshops or courses, and watching movies. Data are presented as percentage of respondents with poor sleep quality (*p* < 0.05 indicates statistical significance, χ2 test).

**Table 4:** Logistic regression analysis for the association between lifestyle factors and poor sleep quality during the COVID-19 confinement.

	*n*	Unadjusted OR	95% CI	*p* Value	Adjusted OR ^1^	95% CI	*p* Value
Age (years)	899	0.989	0.977, 1.002	0.097	0.994	0.980, 1.008	0.422
Sex							
Female	674	1			1		
Male	225	0.603	0.428, 0.851	0.004	0.683	0.471, 0.991	0.045
Sleep duration ^2^							
Inadequate	152	1			1		
Adequate	747	0.505	0.309, 0.824	0.006	0.515	0.304, 0.872	0.014
Chronic comorbidities							
Yes	524	1			1		
No	375	0.577	0.421, 0.791	0.001	0.613	0.435, 0.863	0.005
Tobacco use							
No	730	1			1		
Yes	169	1.646 *	1.054, 2.569	0.028	1.680 *	1.050, 2.688	0.030
Living with a person diagnosed with COVID							
No	841	1			1		
Yes	58	2.185 **	0.976, 4.892	0.057	1.880 *	0.669, 5.280	0.231
Being diagnosed with COVID							
No	861	1			1		
Yes	38	2.522 **	0.884, 7.193	0.084	1.744 *	0.461, 6.600	0.413
Screen time (hours) ^3^							
3 or less	326	1			1		
4 to 7	321	1.411	0.978, 2.037	0.066	1.434	0.967, 2.128	0.073
8 or more	252	1.448	0.975, 2.150	0.067	1.321	0.855, 2.042	0.209
Frequency of late sleep ^4^ (day/week)							
Never	84	1			1		
1 to 2	278	1.570 *	0.947, 2.603	0.080	1.304	0.748, 2.275	0.349
3 to 4	312	3.563 ***	2.097, 6.056	<0.001	2.844 **	1.548, 5.228	0.001
5 to 7	225	3.956 ***	2.235, 7.004	<0.001	2.984 **	1.485, 5.996	0.002
Frequency of late wake up ^5^ (days/week)							
Never	162	1			1		
1 to 2	336	1.502 *	0.987, 2.284	0.057	1.339	0.841, 2.132	0.218
3 to 4	248	1.860 *	1.176, 2.943	0.008	1.165	0.680, 1.995	0.578
5 to 7	153	2.181 **	1.274, 3.733	0.004	1.040	0.534, 2.024	0.908
Change in coffee or black tea intake during lockdown							
No intake	169	1			1		
Considerably reduced	70	1.450	0.708, 2.971	0.310	1.395	0.650, 2.993	0.393
Slightly reduced	41	1.457	0.599, 3.544	0.406	1.157	0.455, 2.938	0.759
Same	388	0.864	0.565, 1.320	0.499	0.815	0.513, 1.295	0.387
Slightly increased	158	1.334	0.779, 2.287	0.94	1.145	0.646, 2.030	0.644
Considerably increased	73	1.264	0.638, 2.505	0.501	0.956	0.462, 1.979	0.903
Change in sunlight exposure during lockdown							
No exposure	257	1			1		
1 to 2 times/week	372	1.148	0.778, 1.694	0.488	1.177	0.778, 1.779	0.440
3 to 4 times/week	164	0.978	0.612, 1.565	0.927	1.272	0.767, 2.111	0.352
5 to 7 times/week	106	0.630	0.380, 1.045	0.074	0.853	0.494, 1.471	0.567
Lifestyle sedentary	269	1			1		
Active	422	0.790	0.535, 1.166	0.235	0.956	0.630	1.450
Very active	208	0.511	0.332, 0.787	0.002	0.696	0.433	1.121

^1^ Adjusted Odds Ratios for all the variables included in the model. ^2^ Sleep duration (h) was adequate or inadequate, according to the National Sleep Foundation recommendation for age. ^3^ Including TV and computer screen time. ^4^ Going to bed later than usual. ^5^ Waking up later than usual. The OR was considered to determine the effect size, categorized as * small (1.5), ** medium (2), and *** large (3) [23].

## Data Availability

The data used in this manuscript are sensitive and are under the custody of the corresponding author. These sensitive data are currently being processed for publication in the article titled “Impact of Confinement for COVID-19 on Sleep Variables: Dataset in the Adult Population of Mexico City” in the journal *Data in Brief*. Therefore, the data will be made available shortly for those interested in consulting them. However, the dataset may be shared with the editorial team for purposes related to the review process of this manuscript.
